# 372. Detection of SARS-CoV-2 RNAemia in Deceased Tissue Donors

**DOI:** 10.1093/ofid/ofab466.573

**Published:** 2021-12-04

**Authors:** Melissa Greenwald, Eduard Grebe, Valerie Green, Alyce Linthurst Jones, Philip Williamson, Michael Busch, Matthew Kuehnert

**Affiliations:** 1 Donor Alliance; Uniformed Services University of the Health Sciences, Chicago, Illinois; 2 Vitalant Research Institute, San Francisco, California; 3 Creative Testing Solutions, Temoe, Arizona; 4 LifeNet Health, Virginia Beach, Virginia; 5 Musculoskeletal Transplant Foundation; Hackensack Meridian School of Medicine, Edison, New Jersey

## Abstract

**Background:**

Tissue donors are evaluated for communicable disease in order to minimize the risk of transmission to recipients. Although there are data suggesting SARS-CoV-2 viremia across a wide spectrum of illness, prevalence in deceased tissue donors and the potential for transplant transmission are unknown.

**Methods:**

Eight tissue banks participated in a retrospective analysis of samples from eligible deceased tissue donors from Oct 2019 through June 2020, one participant in Canada and the remainder located in the United States. All four Census regions of the continental US and all major racial-ethnic groups were represented. EDTA or sodium citrate plasma aliquots were tested in singlicate with the Research Use Only Procleix SARS-CoV-2 Assay on the Procleix Panther System, which uses transcription-mediated nucleic acid amplification (TMA) technology for detection of the SARS-CoV-2 RNA. Plasma (or if unavailable, serum) aliquots were sent to Grifols for an alternate SARS-CoV-2 nucleic acid amplification (NAT) test to verify reactivity and also sent for antibody testing using the emergency use authorization Ortho VITROS Immunodiagnostic Products Anti-SARS-CoV-2 Total test. The VITROS assay uses immunometric technology for qualitative measurement of total antibody (IgG, IgA and IgM) to SARS-CoV-2. The proportion of donors with confirmed RNAemia (i.e., presence of SARS-CoV-2 RNA in plasma or serum) and 95% confidence intervals were computed.

**Results:**

Of 3,455 donor samples with valid final results, 26 (0.76%) were initially positive for SARS-CoV-2 RNA; of these, 3 were confirmed by alternate NAT. Of donor samples collected in 2019 0.00% (95% CI: 0.00%,0.43%) were confirmed RNAemic, while of those collected in 2020, 0.12% (0.04%,0.34%) were confirmed RNAemic. One of 26 initial positive, and none of the three samples confirmed by alternate NAT, tested positive for anti-SARS-CoV-2 Spike antibodies by serology. Infectivity studies are pending on one sample with sufficient available volume.

**Conclusion:**

The rate of SARS-CoV-2 RNAemia in deceased tissue donors is approximately 1 per 1,000, and it is unknown whether this RNAemia reflects the presence of infectious virus. Given these results, the risk of transmission through tissue is most likely to be low.

**Disclosures:**

**Melissa Greenwald, MD**, **Alamo Biologics** (Consultant)**Eurofins VRL Laboratories** (Consultant)**Right Cell Biologics** (Consultant, Consultant Medical Director) **Eduard Grebe, PhD**, **Gilead Sciences** (Consultant)**Sedia Biosciences Corporation** (Consultant, Grant/Research Support)**Vitalant** (Employee) **Alyce Linthurst Jones, PhD**, **LifeNet Health** (Employee) **Matthew Kuehnert, MD**, **American Association of Tissue Banks** (Board Member)**ICCBBA** (Board Member)**Musculoskeletal Transplant Foundation** (Employee)

